# Digital Alerting and Outcomes in Patients With Sepsis: Systematic Review and Meta-Analysis

**DOI:** 10.2196/15166

**Published:** 2019-12-20

**Authors:** Meera Joshi, Hutan Ashrafian, Sonal Arora, Sadia Khan, Graham Cooke, Ara Darzi

**Affiliations:** 1 Chelsea and Westminster Hospital NHS Foundation Trust London United Kingdom; 2 Department of Surgery and Cancer Imperial College London London United Kingdom; 3 Division of Infectious Diseases Imperial College London London United Kingdom

**Keywords:** diagnosis, electronic health records, sepsis, medical order entry systems, outcome assessment (health care)

## Abstract

**Background:**

The diagnosis and management of sepsis remain a global health care challenge. Digital technologies have the potential to improve sepsis care.

**Objective:**

The aim of this paper was to systematically review the evidence on the impact of digital alerting systems on sepsis related outcomes.

**Methods:**

The following databases were searched for studies published from April 1964 to February 12, 2019, with no language restriction: EMBASE, MEDLINE, HMIC, PsycINFO, and Cochrane. All full-text reports of studies identified as potentially eligible after title and abstract reviews were obtained for further review. The search was limited to adult inpatients. Relevant articles were hand searched for other studies. Only studies with clear pre- and postalerting phases were included. Primary outcomes were hospital length of stay (LOS) and intensive care LOS, whereas secondary outcomes were time to antibiotics and mortality. Studies based solely on intensive care, case reports, narrative reviews, editorials, and commentaries were excluded. All other trial designs were included. A qualitative assessment and meta-analysis were performed.

**Results:**

This review identified 72 full-text articles. From these, 16 studies met the inclusion criteria and were included in the final analysis. Of these, 8 studies reviewed hospital LOS, 12 reviewed mortality outcomes, 5 studies explored time to antibiotics, and 5 studies investigated intensive care unit (ICU) LOS. Both quantitative and qualitative assessments of the studies were performed. There was evidence of a significant benefit of digital alerting in hospital LOS, which reduced by 1.31 days (*P*=.014), and ICU LOS, which reduced by 0.766 days (*P*=.007). There was no significant association between digital alerts and mortality (mean decrease 11.4%; *P*=.77) or time to antibiotics (mean decrease 126 min; *P*=.13).

**Conclusions:**

This review highlights that digital alerts can considerably reduce hospital and ICU stay for patients with sepsis. Further studies including randomized controlled trials are necessary to confirm these findings and identify the choice of alerting system according to the patient status and pathological cohort.

## Introduction

### Sepsis Challenges

Sepsis is a major health concern causing significant mortality worldwide [1,2]. Although used to describe a variety of clinical conditions, the most recent recommendations suggest that sepsis should be defined as a “life threatening organ dysfunction caused by a dysregulated host response to infection” [3]. In the United States, the average annual age adjusted incidence is 300-1000 cases per 100,000 people [4]. The mortality from this condition is as high as one in four patients [1,2,5,6]. In addition, it poses a significant financial burden on health care systems. The annual total costs of sepsis in the United States was US $20 billion in 2011 [7]. Additionally, there are numerous indirect costs that may significantly impact patients’ quality of life. For example, the older population may have severe long-term health problems after sepsis, including both cognitive dysfunction and functional disability [8].

### Early Detection of Sepsis

Recognizing sepsis early and initiating timely treatment results in improved patient outcomes and significant cost reduction [9,10]. The International Surviving Sepsis Campaign recommends early identification of patients with sepsis and immediate treatment such as antibiotics within 1 hour of suspecting sepsis and septic shock [11]. A retrospective multicenter study found for every hour’s delay in the treatment of patients with septic shock, the risk of death increases by 7.6% [12]. Delays in identification of sepsis are often attributable to a lack of diagnostic tools, gradual disease progression, and no gold standard for diagnosis [13]. Interventions such as regular monitoring of vital signs and elevated lactate levels aid early recognition [14]. However, despite these measures, patients are still being diagnosed late. Although track and trigger scoring systems such as the National Early Warning Score have standardized the documentation of vital signs and enabled earlier recognition to a degree, they are still subject to inaccuracies in recordings because of their subjective nature and intermittent monitoring [15]. Alternate strategies in sepsis detection are therefore urgently required to improve outcomes.

### Digital Technology in Sepsis

Digital technology holds significant promise in enabling early sepsis recognition. Coupled with the rapid expansion of electronic health records (EHRs) worldwide, it is now possible to provide health care staff with real-time information on laboratory tests, imaging, and physiological vital signs at the bedside. This is particularly relevant in sepsis because of the traditional reliance on risk scoring systems used to define sepsis. These include the Systemic Inflammatory Response Syndrome (SIRS) and Sequential Organ Failure Assessment (SOFA) score [16,17]. Although usually calculated through paper-based observational charts alongside separate interfaces to retrieve blood test results, both have the potential to be automated using EHRs to reduce error and enhance digital alerting. There is now increased literature in this field that can support new clinical decisions in sepsis management. The aim of our paper was to offer an up-to-date systematic review of digital alerting systems on patient sepsis outcomes.

## Methods

### Data Sources and Searches

We applied the Preferred Reporting Items for Systematic Reviews and Meta-analyses (PRISMA) statement for this review [18]. Our search on February 12, 2019, included the following databases: EMBASE, MEDLINE, HMIC, PsycInfo, and Cochrane (April 1964 to February 12, 2019) without language restriction. The search was limited to articles reporting the use of electronic alerts in adult medical inpatients with sepsis who were not initially in intensive care. The search terms included digital technology and sepsis (for complete search strategy, see the Multimedia Appendix 1 search strategy). There were supplemental references that were manually gathered through reference lists.

### Outcome Measures

Our primary outcome was hospital and intensive care unit (ICU) length of stay (LOS) for patients with sepsis. Secondary outcome measures included sepsis-associated mortality and the time to antibiotics.

### Study Selection

Studies were eligible for inclusion if patients had sepsis with a clear reporting of the diagnostic criteria. In addition, studies were only eligible for inclusion if the outcomes could be compared pre- and postintroduction of digital alerts for sepsis. Studies where the clear outcome measures of interest were not stated were not included. The outcomes of interest were mortality, time to antibiotics, and ICU LOS, and hospital LOS. Studies only required one outcome of interest to be eligible for inclusion. There was no fixed number of patients required in each treatment group nor a fixed duration of follow-up.

### Data Extraction and Quality Assessment

All included study characteristics and outcome measures were extracted by one investigator and verified by another. All full-text reports of studies identified as potentially eligible after title and abstract review were obtained for further review. All empirical research into digital sepsis alerting in adults and outcome measures were included. Information on the study location, type of hospital, type of alert, and whom the alert was directed at were all extracted using a set standardized form. Studies were only included if a comparative study of pre- and postalerting groups was performed and the number of patients in each group was clearly stated. Studies solely based on intensive care were excluded, as extra levels of care and treatments offered to these patients would affect the sepsis outcome measures. Additionally, case reports, narrative reviews, editorials, and commentaries were excluded. After full text review, if a study was excluded, the reasons were noted and disagreements were resolved by a third reviewer. A PRISMA flow diagram was used to map studies included, studies excluded, and the reasons for their inclusion/exclusion (Multimedia Appendix 2).

### Data Synthesis and Analysis

A qualitative assessment of all studies was performed. For the primary outcome of hospital LOS, a weighted mean difference was calculated. A weighted mean difference was also used in ICU LOS. For the secondary outcome measure of mortality, we used percentage change between the pre- and postalerting groups. To ensure a standard way to compare the studies, all times to antibiotics were converted into minutes and number of days used for ICU/hospital LOS. For the meta-analysis, outcomes were analyzed by calculating the difference in means between studies or the ratio of means within each study. We substituted the median for the mean in studies where only the median was reported. We employed an inverse-variance approach with a random effects model using the DerSimonian and Laird methodology for both continuous and categorical variables. This was accomplished using Stata 13 (StataCorp, College Station, Texas). The *I*^2^ statistic was used to estimate the degree of heterogeneity between studies.

### Risk-of-Bias Assessment

The Newcastle-Ottawa scale (NOS) was used to score the included papers on the basis of the quality [19]. The range of scores was from 0 to 9. Studies with a score of ≥7 were rated as *higher-quality* studies, whereas those with scores ≤ 7 were rated as *lower-quality* studies.

## Results

### Search Results

The literature search identified 3861 references (appendix). One article was found through hand searching. After screening, 72 full-text reviews were reviewed, including one translated from Spanish. A total of 16 studies met the inclusion criteria and were included in the final analysis. A description of the studies including the sepsis intervention, alert type, and criteria used to define sepsis can be found in Multimedia Appendix 3. All studies that defined sepsis alerts and the pre- and postalerting stages are presented in Multimedia Appendix 4.

### Characteristics of Studies and Digital Alerting Used

All studies investigated digital sepsis alerts (Multimedia Appendix 3). Several studies additionally used a rapid response team in combination with the digital sepsis alert in the alerting group [20-22]. An electronic order set was used by four studies in the alerting group [21,23-25].

The studies were predominantly single-center studies; however, one study that was multicenter study across three hospital sites [26]. Most studies were based in the United States; however, one study was from the Kingdom of Saudi Arabia [20] and one was from Spain [27]. Most studies were based in large hospital settings; six studies were based in academic centers [20,22,23,28-30], and three centers were level 1 trauma centers [21,29,31]. Within the hospital setting, only one study had a hospital-wide sepsis alert [21], and eight had alerts solely in the emergency department (ED) [20,23,27,29-33]. In addition, five studies included only medical wards [24,26,28,34,35], and two studies included both on medical and surgical wards [22,25]. All the studies were pre- and postimplementation studies.

The type of electronic alerts used in the studies varied. Most studies used digital alerts generated through EHRs on desktop-based computers [20-23,27,29-35]. Two studies involved alerting via a mobile device [24,25] and two studies used text paging [26,28]. One study predominantly used alerting through EHRs but used additional text paging at the discretion of the ED attending [23]. This is shown in Multimedia Appendix 3. Most of the digital alerts in the study were aimed at the nursing staff [20,22-25,28,32]. Although one study was aimed solely at clinicians [31], two studies had alerts sent to both clinicians and the rapid response team [21,26]. In addition, in four studies, it was unknown whom the digital alerts were sent to [27,29,33,35].

The sepsis-alerting criteria varied among studies; many studies used a variation of the SIRS criteria [20-24,26,29,34]. SIRS is defined as a temperature >38°C or <36°C, heart rate >90 beats/min, respiratory rate >20 breaths/min or arterial CO_2_ lower than 32 mm Hg, and white blood cell coun*t* >12,000/mm^3^ or <4000/mm^3^ [16]. Only one study used two or more SIRS criteria alone [34]. Some used two or more SIRS criteria with a suspicion of infection [21,23,31]. Hayden et al used two or more SIRS criteria with a suspicion of infection but had two subgroups of patients: one with a systolic blood pressure (SBP) of <90 mm Hg and one with an SBP >90 mm Hg. A combination of two or more SIRS criteria with evidence of end-organ dysfunction was also used as a criterion to alert [20,26,30]. Narayanan et al reported two types of sepsis alerts: one alert if two or more SIRS criteria were used and another if severe sepsis/septic shock criteria (ie, two or more SIRS criteria + end-organ dysfunction OR fluid nonresponsive hypotension) [30]. One study used the combination of two or more SIRS criteria and anion gap acidosis [22]. Crum et al used a combination of two or more SIRS criteria; infectious ED diagnosis or symptom diagnosis and antibiotic administration in the ED; and acute organ dysfunction (lactate level ≥4.0 mmol/L and persistent hypotension within 6 hours of triage or vasopressor use) [29]. Austrian et al had two types of alerts: one with two or more SIRS criteria and another with an SBP <90 mm Hg or lactate level ≥ 4 mg/dL. Not all studies used SIRS; Pulia et al [33] used a fever plus any abnormal vital sign, whereas Sawyer et al [28] and Ferreras et al [27] used an algorithm consisting of laboratory values and hemodynamic parameters. The study by Mathews et al [35] was an abstract only and no details were found on the alerting criteria used.

All studies included had clear outcome measures with a defined sepsis alert and pre- and postalerting stage (Multimedia Appendix 4). Some studies reviewed several outcome measures of interest. All studies were non-ICU based and either alerted in the ED or the hospital wards. In total, 8 studies reviewed the hospital LOS, 5 studies reviewed the ICU LOS, 11 reviewed the mortality outcomes, and 5 studies reviewed the time to antibiotics. Several studies have tried to adjust for potential confounders. Guirgis et al [21] performed multivariate adjusted comparisons, whereas Westra [25] used techniques such as propensity scoring and bootstrapping to adjust for confounders [25]. Each outcome measure is reviewed below in further detail.

### Hospital Length of Stay

Hospital LOS was included in eight studies [20,21,23,25,26,28,32,34]. In total, there were 3948 patients in the prealerting group and 4872 patients in the postalerting group across these eight studies. The results highlight a significant reduction in hospital LOS by 1.311 days in the postalerting group (95% CI –2.362 to –0.261; *P*=.014). The proportional decrease in hospital LOS was the study with the biggest impact on LOS, which was Arabi et al [20]. The strong treatment effect seen may be partly explained by the addition of a rapid response team; however, the rapid response team was also used in the study by Guirgis et al [21] and the effect on LOS was not as remarkable.

### Intensive Care Unit Length of Stay

For ICU LOS (when patients were escalated from the ward setting), five studies were included [20,21,23,26,32] (Multimedia Appendix 4). In total, 3627 patients were included in the prealerting group and 4475 were included in the postalerting group. The weighted mean difference for all studies showed a significant reduction in ICU LOS by 0.766 days for the alerting group versus the prealerting group (95% CI –1.324 to 0.209; *P*=.007). The biggest impacting study was the one by Arabi et al [20]. This may have been explained by the addition of a rapid response team; however, the rapid response team was also used in the study by Guirgis et al [21] and the effect on LOS was not as remarkable.

### Mortality

For mortality, 11 studies were included [20,24,27,28,31,32,34,35]. In total, there were 5868 patients in the prealerting group and 6629 in the postalerting group. There was no significant reduction between digital alerts and mortality (mean decrease 11.4%; 95% CI –0.873 to 0.646; *P*=.77).

### Time to Antibiotics

For time to antibiotics, five studies were included [20,23,29,33] (Multimedia Appendix 4). In total, there were 991 patients in the prealerting group and 1473 in the postalerting group. On the basis of three studies with available data, the weighted mean difference in time to antibiotics from the prealerting to the alerting group showed no significant reduction (126 min) in time to antibiotics (95% CI -291.113 to 39.015; *P*=.13).

### Diagnostic Accuracy

The diagnostic accuracy of the digital sepsis alerts is shown in Multimedia Appendix 5. Of all the abovementioned studies included in the review, only five studies attempted to address the diagnostic accuracy [24,32]. There was high heterogeneity in the accuracy of alerting in these studies. The specificity of digital alerting in sepsis remained high, with four studies reporting specificities of 81.92% (78.73-84.8 range) [24], 97% [26], 82.0% [25], and 94.56% (93.64-95.32 range) [27]. Although the specificity remained high, the sensitivity showed high heterogeneity: 95.16% (89.77-98.20) [24], 95.2% [25], 87% (81.93-91.66) [27], 80.4% [32], and 16% [26]. The sensitivities and specificities were not given in any other studies. In total, three studies had high negative predictive values of digital alerting and sepsis ranging from 94% [26] to 98.88% (97.58-99.59) [24] and 99.11% (98.69-99.4) [27]. The positive predictive value was much lower at 14.6% [32], 26% [26], 50.21% (43.64-56.78) [24], and 51.64% (46.15-57.11) [27].

### Risk of Bias Assessment

All included studies were assessed for the quality of their methodology. All studies had a nonrandomized design methodology, and the NOS was used (Multimedia Appendix 6). Of all studies included, only six were of *higher* quality. Many had poor comparability due to a lack of randomization.

## Discussion

Patients with sepsis have high morbidity, mortality, and associated treatment costs [1,7,36].

This review found that digital alerting in sepsis is associated with significant reductions in hospital LOS by 1.311 days (*P*=.014) and ICU LOS by 0.766 days (*P*=.007). Both mortality and time to antibiotics showed no significant differences between the prealerting and postalerting groups.

Reduced hospital LOS and ICU LOS are likely due to earlier diagnosis, improved time to treatments such as antibiotics, earlier fluid replacement, minimized inflammatory cytokines, and cytokine stress. A previous systematic review on the diagnostic accuracy and effectiveness of digital alerts found no improvements in clinical outcomes when digital alerts in sepsis were employed [37]. However, since its publication, there has been a growth in the number of empirical studies on digital alerting and sepsis outcomes, as more hospitals worldwide are transferring to EHRs and digital solutions. All studies identified in this review are recent and after 2010, reflecting the entry of new technology in this field.

This review also identified a wide variation in the diagnosis of sepsis and multiple measures used for this purpose. Many of the studies included in the review have used the SIRS definition of sepsis or a modification of it. This is perhaps because it is the most practical in a clinical setting with readily available vital sign data and laboratory tests. Although SIRS was routinely used as a definition of sepsis, the Third International Consensus Definitions for Sepsis and Septic Shock (Sepsis 3) declared that the use of two or more SIRS criteria in identifying sepsis was not specific enough [3]. The SIRS criteria may be met without infective disease processes, such as trauma [38]. The variation in diagnostic criteria may explain the lack of literature meta-analysis in digital sepsis alerts to date. The ideal alerting criteria required for sepsis in combination with guidance on the latest definitions must be reviewed in further large-scale randomized studies. The ideal sepsis alerts must correctly identify patients with sepsis and exclude those without sepsis. Alerting with low positive predictive value is likely to contribute to alert fatigue, and it is suggested that more sophisticated algorithms may be required to correctly identify patients with sepsis [32]. A recent retrospective comparison of scoring systems found the National Early Warning Score to be more accurate in detecting sepsis as compared with other scoring systems including SIRS [39]. Furthermore, multicenter studies are required to review these findings. In addition to the alert itself, the way the alert is delivered must be reviewed. There is no one best method in the literature to the type of alert to be used and who it needs to be sent to. A combination of EHR, text paging, and mobile alerts have been used in the studies included in this review.

Of all the studies included in the review, only five studies attempted to address diagnostic accuracy [24,32]. There was high heterogeneity in the accuracy of the alerting in these studies. Far more research is required in large studies to elucidate diagnostic accuracy in digital alerting. Although digital alerting in sepsis is beneficial, it is likely that the alerts be used in combination with an application of other resources such as staff education, direct communication for patients with sepsis, and rapid response teams. In one study, 5 months of training in addition to the sepsis alert helped contribute to a reduction in sepsis mortality [24].

Despite the significance of some of the clinical outcomes assessed, our study has limitations due to the variability of methodologies and study types, and most studies scored low on quality. Most of the studies are observational in design, thus creating a high risk of bias. There was a high heterogeneity between the studies, and there were very limited data on the diagnostic accuracy of many of the included results. Furthermore, the pre- and postalerting study design may be prone to selection bias due to variations in the patient population and changes in ways of providing care at hospitals over time. A further limitation of the studies is that the size of the postalerting cohort is larger than that of the prealerting cohort, despite the fact that many of the included studies followed postimplementation cohorts for a shorter period of time than the preimplementation cohort. Although all studies used electronic digital alerting, some studies additionally used a rapid response team and electronic order sets. This may have led to a confounding bias in outcomes.

There are several studies published recently with different study designs to help address some of these concerns. The first is a retrospective cohort study with patients exposed or not exposed to the electronic ICU telemedicine sepsis management in the ED [40]. This is a novel form of alerting using telemedicine. In this study, the exposed cohort had a quicker time to antibiotics than the unexposed cohort (122.3 [SD 83.3] min versus 163.4 [SD 204.4] min; *P*=.04] [40]. However, the hospital LOS and mortality were similar between both cohorts.

Another study design is the use of a prospective quality improvement study [41]. The alerting system was customized to local practice with regularly incorporated feedback through the plan-do-study-act (PDSA) cycles [41]. In this study, a machine learning algorithm was implemented with continuous incorporation of feedback, creating a system tailor made to the hospital workflow [41]. After several PDSA cycles, the machine learning algorithm sepsis mortality reduced by 60.24% with a hospital LOS reduction of 9.55% [41]. The alerting in this study was very different from that in other studies because of the machine learning approach [41]. Although it was not included in the original meta-analysis, the analysis was redone to include this study, and there was no difference in either the hospital LOS (proportional decrease: 11.3%; 95% CI –0.189 to 0.038; *P*=.003) or mortality (mean decrease: 11.4%; 95% CI –0.844 to 0.616; *P*=.76).

Further research should also seek to answer several other important questions such as the optimal type of sepsis alert, which team members should be alerted, and at what frequency should they be alerted. A recent observational cohort study reviewing successive improvements over a 10-year period found that reviewed sepsis alerts were sent to a telephonist and alerts were sent to a nurse’s mobile phone [42]. They found that time to antibiotics was reduced to 1 hour (55 min to 1 hour 30 min) when the alert was sent to the telephonist and to 45 min (30 min to 1 hour) when the alert was sent directly to the nurse’s mobile phone (*P*=.02) [42].

Further large-scale multicenter prospective randomized controlled trials (RCTs) with a single standardized sepsis alert are required. An RCT would help exclude confounding biases, although implementation maybe challenging. Overall, two recent RCTs have been published; however, both studies had insufficient sample sizes, with insufficient power to detect differences between the prealerting and postalerting groups [43,44]. The results of both RCTs were also contradictory. One RCT on sepsis alerting through EHRs found no significant difference in outcome measures after alerting [43]. However, at least 66% of patients were on antibiotics at the time of the alert, and this high baseline compliance may have led to a lower marginal return on improved detection through alerting and partly explain these findings [43]. The other RCT on the use of machine learning–based sepsis prediction systems supported similar findings to this review with a reduction in hospital LOS by 2.7 days and a mortality reduction of 58% [44].

The cumulative effect of an additional rapid response team, staff education, and electronic order sets alongside digital alerting must also be evaluated. Further studies should also be performed in different clinical health systems, as the value of alerting is likely to depend on baseline performance of health systems. This would aid generalizability and widespread implementation. Importantly, the diagnostic accuracy for the digital alerting in most studies included is unknown and needs to be clearly defined in future work to ascertain the validity of findings. Finally, the cost-effectiveness of digital sepsis alerts also needs further evaluation if we are to justify its use in the current fiscal climate.

Automated digital alerts can improve sepsis-related outcomes. This review highlights a significant reduction in hospital LOS by 1.311 days and ICU LOS by 0.766 days. The emergence of digital technologies has the capacity to transform the rapid identification of patient physiological deterioration and, in turn, revolutionize patient care through alerts and novel treatment innovations (such as *smart wards)*. Higher-quality evidence through larger, better-designed randomized studies is needed to guide the application of digital alerting in patients at risk of sepsis.

## Appendix

Multimedia Appendix 1 Search strategy.

Multimedia Appendix 2 Preferred Reporting Items for Systematic Reviews and Meta-analyses flow diagram.

Multimedia Appendix 3 Characterization of studies and digital alerts used.

Multimedia Appendix 4 All studies reviewing outcome measures.

Multimedia Appendix 5 Diagnostic accuracy.

Multimedia Appendix 6 Risk of bias of a nonrandomized prospective comparative cohort comparing pre and postalerting based on the Newcastle-Ottawa Scale.

## Abbreviations

BRC: Biomedical Research Centre
ED: emergency department
EHR: electronic health record
ICU: intensive care unit
LOS: length of stay
NOS: Newcastle-Ottawa scale
NIHR: National Institute for Health Research
PDSA: plan-do-study-act
PRISMA: Preferred Reporting Items for Systematic Reviews and Meta-analyses
PSTRC: Patient Safety Translational Research Centre
RCT: randomized controlled trial
SBP: systolic blood pressure
SOFA: sequential organ failure assessment
SIRS: systemic inflammatory response syndrome
