# Supplementary material for: Digital Alerting and Outcomes in Patients With Sepsis: Systematic Review and Meta-Analysis
Source: J Med Internet Res. 2019 Dec 20;21(12):e15166. doi: 10.2196/15166 (PMC6942184; doi:10.2196/15166)
Supplement: Multimedia Appendix 1 [file jmir_v21i12e15166_app1.docx]

Multimedia Appendix 1 Search Strategy

The search was performed on 12/02/2019.

Medline Search 596 [plus 148] = 744

| 1 | digital platform* or electronic platform* or electronic alert* or [Electronic or automated or digital] adj 2 [record* or system*]] or machine learning* or AI* or Artificial Intelligence* or [[ Electronic or automated or digital] and escalation] or Machine learning [MESH term] or Supervised machine learning [MESH term] or Decision theory [MESH term] or medical informatics [MESH term] or health information exchange [MESH term] or medical informatics applications [MESH term], decision making, computer –assisted [MESH term] or diagnosis, computer assisted [MESH term] or therapy, computer assisted [MESH term] or telemedicine [MESH term] or telepathology [MESH Term] or eHealth* or mhealth* or technology [MESH term] or internet [MESH term] or communications media [MESH term] or programmed as topic [MESH term] or computers, handheld [MESH term] or smartphone [MESH term] or mobile applications [MESH term] or cellphones [MESH term] or text messaging [MESH term] or [[mobile* or cell* or smart*] and phone*] or [digital adj health]*   \|  \| \| --- \| |
| --- | --- | --- |
| 2 | 1 or 2 or 3 or 4 or 5 or 6 or 7 or 8 or 9 or 10 or 11 or 12 or 13 or 14 or 15 or 16 or 17 or 18 or 19 or 20 or 21 or 22 or 23 or 24 or 25 or 26 or 27 or 28 or 29 or 30 or 21 or 32 |
| 3 | Sepsis [MESH term] or bacteremia [MESH term] or fungemia [MESH term] or shock, septic [MESH term], or sepsis* or septicaemia* or Systemic inflammatory response syndrome [MESH term] or systemic inflammatory response syndrome* or SIRS* or septic shock* |
| 4 | 1 or 2 or 3 or 4 or 5 or 6 or 7 or 8 or 9 or 10 |
| 5 | 2 and 4 |

Embase search 2512 +420 = 2932

| 1 | digital platform* or electronic platform* or electronic alert* or [Electronic or automated or digital] adj 2 [record* or system*]] or machine learning* or AI* or Artificial Intelligence* or [[ Electronic or automated or digital] and escalation] or Machine learning [SUBJECT HEADING] or data mining [SUBJECT HEADING], or supervised machine learning [SUBJECT HEADING] or medical informatics [SUBJECT HEADING] or artificial intelligence [SUBJECT HEADING] or telemedicine [SUBJECT HEADING] or telepathology [SUBJECT HEADING] or eHealth* or mhealth* or technology [SUBJECT HEADING] or internet [SUBJECT HEADING] or personal digital assistant [SUBJECT HEADING] or smartphone [SUBJECT HEADING] or mobile applications [SUBJECT HEADING] or mobile phone [SUBJECT HEADING] or text messaging [SUBJECT HEADING] or [[mobile* or cell* or smart*] and phone*] or [digital adj health]*   \|  \| \| --- \| |
| --- | --- | --- |
| 2 | 1 or 2 or 3 or 4 or 5 or 6 or 7 or 8 or 9 or 10 or 11 or 12 or 13 or 14 or 15 or 16 or 17 or 18 or 19 or 20 or 21 or 22 or 23 or 24 or 25 or 26 |
| 3 | Sepsis [SUBJECT HEADING] or Systemic inflammatory response syndrome [SUBJECT HEADING] or bacteremia [SUBJECT HEADING] or fungemia [SUBJECT HEADING] or septic shock [SUBJECT HEADING], septicaemia [SUBJECT HEADING] or sepsis* or systemic inflammatory response syndrome* or SIRS* or septic shock* |
| 4 | 1 or 2 or 3 or 4 or 5 or 6 or 7 or 8 or 9 or 10 |
| 5 | 2 and 4 |

Health Management Information Consortium [HMIC] Search 4+1=5

| 1 | digital platform* or electronic platform* or electronic alert* or [Electronic or automated or digital] adj 2 [record* or system*]] or machine learning* or AI* or Artificial Intelligence* or [[ Electronic or automated or digital] and escalation]* or decision theory [SUBJECT HEADING] or medical informatics [SUBJECT HEADING] or electronic patient records [SUBJECT HEADING] or medical information exchange [SUBJECT HEADING] or medical informatics [SUBJECT HEADING] or computer aided learning [SUBJECT HEADING] or artificial intelligence [SUBJECT HEADING] or telemedicine or medical telemetering equipment [SUBJECT HEADING] or telemetry [SUBJECT HEADING] or digital technology [SUBJECT HEADING] or internet [SUBJECT HEADING] or electronic data exchange [SUBJECT HEADING] or personal computers. Or laptop computers [SUBJECT HEADING] personal digital assistant [SUBJECT HEADING] or tablet personal computer [SUBJECT HEADING] or mobile communication systems [SUBJECT HEADING] or mobile telephones [SUBJECT HEADING] or text messaging [SUBJECT HEADING] or [[mobile* or cell* or smart*] and phone*] or [digital adj health]*   \|  \| \| --- \| |
| --- | --- | --- |
| 2 | 1 or 2 or 3 or 4 or 5 or 6 or 7 or 8 or 9 or 10 or 11 or 12 or 13 or 14 or 15 or 16 or 17 or 18 or 19 or 20 or 21 or 22 or 23 or 24 or 25 or 26 or 27 or 28 or 29 or 30 |
| 3 | Sepsis [SUBJECT HEADING] or sepsis* or septicaemia or septic shock or Systemic inflammatory response syndrome or SIRS |
| 4 | 1 or 2 or 3 or 4 or 5 or 6 |
| 5 | 2 and 4 |

Psych Info Search 98+11=109

| 1 | digital platform* or electronic platform* or electronic alert* or [Electronic or automated or digital] adj 2 [record* or system*]] or machine learning* or AI* or Artificial Intelligence* or [[Electronic or automated or digital] and escalation]* or machine learning [SUBJECT HEADING] or artificial intelligence [SUBJECT HEADING] or decision theory [SUBJECT HEADING] or telemedicine [SUBJECT HEADING] or computer assisted diagnosis [SUBJECT HEADING] or computer assisted therapy [SUBJECT HEADING] or eHealth* or Mhealth* or Technology [SUBJECT HEADING] or internet or computer applications or communications media or cellular phones [SUBJECT HEADING] or mobile devices [SUBJECT HEADING] or text messaging or [[mobile* or cell* or smart*] and phone*] or [digital adj health]*   \|  \| \| --- \| |
| --- | --- | --- |
| 2 | 1 or 2 or 3 or 4 or 5 or 6 or 7 or 8 or 9 or 10 or 11 or 12 or 13 or 14 or 15 or 16 or 17 or 18 or 19 or 20 or 21 or 22 or 23 or 24 or 25 |
| 3 | Sepsis* or septic shock or bacteremia* or fungemia* or septicaemia* or Systemic inflammatory response syndrome or SIRS* |
| 4 | 1 or 2 or 3 or 4 or 5 or 6 or 7 |
| 5 | 2 and 4 |

Cochrane 2 + 69 = 71

| 1 | digital platform* or electronic platform* or electronic alert* or [Electronic or automated or digital] adj 2 [record* or system*]] or machine learning* or AI* or Artificial Intelligence* or [[ Electronic or automated or digital] and escalation] or Machine learning [MESH term] or Supervised machine learning [MESH term] or Decision theory [MESH term] or medical informatics [MESH term] or health information exchange [MESH term] or medical informatics applications [MESH term], decision making, computer –assisted [MESH term] or diagnosis, computer assisted [MESH term] or therapy, computer assisted [MESH term] or telemedicine [MESH term] or telepathology [MESH Term] or eHealth* or mhealth* or technology [MESH term] or internet [MESH term] or communications media [MESH term] or computers, handheld [MESH term] or smartphone [MESH term] or mobile applications [MESH term] or text messaging [MESH term] or [[mobile* or cell* or smart*] and phone*] or [digital adj health]*   \|  \| \| --- \| |
| --- | --- | --- |
| 2 | 1 or 2 or 3 or 4 or 5 or 6 or 7 or 8 or 9 or 10 or 11 or 12 or 13 or 14 or 15 or 16 or 17 or 18 or 19 or 20 or 21 or 22 or 23 or 24 or 25 or 26 or 27 or 28 or 29 or 30 or 21 or 32 |
| 3 | Sepsis [MESH term] or bacteremia [MESH term] or fungemia [MESH term] or shock, septic [MESH term], or sepsis* or septicaemia* or Systemic inflammatory response syndrome [MESH term] or systemic inflammatory response syndrome* or SIRS* or septic shock* |
| 4 | 1 or 2 or 3 or 4 or 5 or 6 or 7 or 8 or 9 or 10 |
| 5 | 2 and 4 |
