# Supplementary material for: Digital Alerting and Outcomes in Patients With Sepsis: Systematic Review and Meta-Analysis
Source: J Med Internet Res. 2019 Dec 20;21(12):e15166. doi: 10.2196/15166 (PMC6942184; doi:10.2196/15166)
Supplement: Multimedia Appendix 2 [file jmir_v21i12e15166_app2.docx]

*Figure 1 PRISMA Flow Diagram*

Studies included in quantitative synthesis.

( meta-analysis )
( n=16 )

Studies included in qualitative synthesis.
( n=16 )

Full-text articles assessed for eligibility.
( n=72 )

Records excluded
(n=3282)

Records after duplicates were removed.

( n=3354 )

Records identified through database searching.

( 3861 )

Additional records identified through other sources.
( n=1 )

Identification

Screening

All Records that were screened.
( n=3354 )

Full-text articles and abstracts excluded, with reasons

( n=56 )

Electronic order set / Decision support tool not sepsis alert: 3

No real pre/post alert stage: 11

Predicting model of mortality / Antibiotics: 3

Time to Antibiotics not given: 3

Mortality not given, survival stated: 1

Not a specific alert for sepsis: 4

ICU based study: 5

Outcomes of interest not measured: 8

Mortality number of patients / percentage not given: 10

Patient numbers not clearly stated: 2

Alert generated but not sent to clinical team: 2

Pediatrics: 1

No outcome data: 3

Eligibility

Included
