# Supplementary material for: Digital Alerting and Outcomes in Patients With Sepsis: Systematic Review and Meta-Analysis
Source: J Med Internet Res. 2019 Dec 20;21(12):e15166. doi: 10.2196/15166 (PMC6942184; doi:10.2196/15166)
Supplement: Multimedia Appendix 3 [file jmir_v21i12e15166_app3.docx]

Multimedia Appendix Table 1. Characterization of studies and digital alerts used.

| Study | Intervention | Period | Alert type | Digital sepsis alert criteria |
| --- | --- | --- | --- | --- |
| Arabi et al, 2017 A [20], Kingdom of Saudi Arabia | Group 1 is sepsis alert alone | - Preimplementation = 20 months - Postimplementation (Phase 1 = 6 months; Phase 2 = 7 months) - Total duration = 33 months | Sepsis electronic alert to nursing worklist (desktop based via EHRs) and CPOE sepsis management order set | A combination of SIRS and 1 organ dysfunction. >2 SIRS criteria AND at least one of the following organ dysfunctions (SBP 86-90 mmHg with intravenous fluids or <86 mmHg regardless of fluids; blood oxygen saturation of 85%-90% with supplemental oxygen, or <85% without oxygen; lactate >2 mmol/L) OR 2 of the above organ dysfunctions |
| Arabi et al, 2017 B [20], Kingdom of Saudi Arabia | Group 2 is sepsis alert and the rapid response team (ICU registrar and trained sepsis nurse) | - Preimplementation = 20 months - Postimplementation (Phase 1 = 6 months; Phase 2 = 7 months) - Total duration = 33 months | Sepsis electronic alert to nursing worklist (desktop based via EHRs) and CPOE sepsis management order set | A combination of SIRS and 1 organ dysfunction. >2 SIRS criteria AND at least one of the following organ dysfunctions (SBP 86-90 mmHg with intravenous fluids or <86 mmHg regardless of fluids; blood oxygen saturation of 85-90% with supplemental oxygen, or <85% without oxygen; lactate >2 mmol/L) OR 2 of the above organ dysfunctions |
| Austrian et al, 2018 [32], New York, United States | Sepsis alert | - Preimplementation = 12 months - Postimplementation = 14 months - Total duration = 28 months | Alert on electronic medical record (desktop based via EHRs) to nurses and clinical sepsis champions | SIRS advisory alert = >2 SIRS criteria or sepsis advisory alert = SBP <90 mmHg or lactate >4 mg/dl |
| Benson et al, 2014 [22], Michigan, United States | Sepsis alert and nurse practitioner rapid response team | - Preimplementation = 12 months - Postimplementation = 6 months - Total duration = 18 months | Alert to nurse practitioner rapid response team (desktop based) | >2 SIRS criteria + anion gap acidosis |
| Berger et al, 2010 [31], New York, United States | Sepsis alert | - Preimplementation = 6 months - Postimplementation = 6 months - Total duration = 12 months | Clinical decision support electronic alert (desktop based via EHRs) to clinicians | >2 SIRS criteria and physician suspicion of infection |
| Crum et al, 2013 [29], California, United States | Sepsis alert | - Preimplementation = 7 months - Postimplementation = 7 months - Total duration = 14 months | CDSS alert (desktop based via EHRs) unknown to whom alerts were sent | >2 SIRS criteria; infectious ED diagnosis OR symptom diagnosis and antibiotic administration in the ED; AND acute organ dysfunction (lactate >=4.0 mmol/L, persistent hypotension within 6 hours of triage or vasopressor use) |
| Ferreras et al, 2015 [27], Aragon, Spain | Sepsis alert | - Preimplementation = 12 months - Postimplementation = Unknown - Total duration = Unknown | Alert on electronic medical record (desktop based via EHRs) unknown to whom alerts were sent | Abnormal vital signs (SBP<90mmHg, HR>90, Temperature >38 °C or <36 °C, RR > 30/min, oxygen saturation < 90%, hyperglycemia 140 mg/dl in the absence of diabetes). 2 types of alert. 1 sepsis alert if any 2 parameters an alert sent. Serious alert if any 2 parameters but one is hypotension OR any 3 parameters abnormal |
| Guirgis et al, 2017 [21], Jacksonville, United States | Sepsis alert and rapid response team and educational program and electronic order set | - Preimplementation = 13 months - Postimplementation = 12 - Total duration = 25 months | Alert on electronic medical record (desktop based via EHRs) to clinicians and rapid response team | >2 SIRS criteria and documented source of infection |
| Hayden et al, 2016 A [23], South Carolina, United States | Sepsis alert, direct communication, mobilization of resources and order sets (duration 22 months) | - Preimplementation = 4 months - Postimplementation = 18 months - Total duration = 22 months | Alert on electronic medical record to nurses and paging at the discretion of ED attending (desktop based via EHRs) | SWAT A - SBP <90 mmHg and any other SIRS criteria or suspected or known infection |
| Hayden et al, 2016 B [23], South Carolina, United States | Sepsis alert, direct communication, mobilization of resources and order sets (duration 22 months) | - Preimplementation = 4 months - Postimplementation = 18 months - Total duration = 22 months | Alert on electronic medical record to nurses and paging at the discretion of ED attending (desktop based via EHRs) | SWAT B - SBP>90 mmHg and ≥2 SIRS criteria and concern for an infection source |
| Manaktala et al, 2017 [24], Minneapolis, United States | Sepsis alert, staff education on sepsis screening program, creating sepsis order sets | - Preimplementation = 32 months - Postimplementation = 9 months - Total duration = 41 months | Alert sent to nursing staff’s mobile device | SIRS criteria and infection (comorbidities or medications that alter parameters, such creatinine assessed differently in renal failure patients). If no, then alert, if comorbidities or medications alter parameters, adjust parameter cutoffs based on comorbid conditions |
| Mathews et al, 2014 [35], California, United States | EHR-based clinical decision tool | - Preimplementation = 12 months - Postimplementation = 11 months - Total duration = 23 months | Alert on electronic medical record, automated clinical decision tool (desktop based via EHRs) unknown to whom alerts were sent | Automated clinical decision tool (only abstract, no further details) |
| McRee et al, 2017 [34], Arizona, United States | Sepsis alert | - Preimplementation = 6 months - Postimplementation = 6 months - Total duration = 12 months | Alert on electronic medical record to nurses (desktop based via EHRs) | >2 SIRS criteria |
| Narayanan et al, 2016 [30], San Francisco, United States | Sepsis alert | - Preimplementation = 7 months - Postimplementation = 7 months - Total duration = 14 months | Alert on electronic medical record to health care providers (desktop based via EHRs) | 2 EMR alerts: 1 alert if ≥2 SIRS criteria and another if severe sepsis/septic shock criteria (ie, ≥2 SIRS criteria and end-organ dysfunction OR fluid nonresponsive hypotension) |
| Pulia et al, 2016 [33], Madison, United States | Sepsis alert | - Preimplementation = unknown - Postimplementation = unknown - Total duration = unknown | Alert on electronic medical record (desktop based via EHRs), unknown to whom alerts were sent | Fever and an additional abnormal vital sign |
| Sawyer et al, 2011 [28], Washington, United States | Sepsis alert | - Preimplementation = unknown - Postimplementation = unknown - Total duration = 8 months | Text page to nurse in charge of the ward | Algorithm consisting of laboratory values and hemodynamic parameters including the shock index (HR/SBP), mean arterial pressure, international normalized ratio, white blood cell count, hemoglobin, absolute neutrophil count, serum albumin, total bilirubin, and sodium |
| Umscheid et al, 2015 [26], Philadelphia, United States | Sepsis alert | - Preimplementation = 3 months - Postimplementation = 3 months - (1 year after alert was implemented) Total duration =6 months | Alert to the covering provider and rapid response coordinator by text page. The bedside nurses had an alert on electronic medical record | SIRS and organ dysfunction (SBP < 100 mm Hg + serum lactate measure >2.2 mmol/L) |
| Westra et al, 2017 [25] Minneapolis, United States | Sepsis alert | - Preimplementation = 4 months - Postimplementation = 10 months - Total duration = 14 months | Alert sent to nurse’s mobile device, order sets, staff education and setting up an interdisciplinary governance committee | Alerting based on 2012 Surviving sepsis campaign and critical care surviving sepsis campaign guidelines. Alerts were disease and medication specific to account for abnormal laboratory tests and vital sign abnormalities |

Footnotes

Arabi A = Group 1 is sepsis alert alone

Arabi B = Group 2 is sepsis alert and the rapid response team (ICU registrar and trained sepsis nurse)

COPE = Computerised Physician Order Entry

EHR = Electronic Health Records
