# Supplementary material for: Digital Alerting and Outcomes in Patients With Sepsis: Systematic Review and Meta-Analysis
Source: J Med Internet Res. 2019 Dec 20;21(12):e15166. doi: 10.2196/15166 (PMC6942184; doi:10.2196/15166)
Supplement: Multimedia Appendix 4 [file jmir_v21i12e15166_app4.docx]

Table 2. All studies reviewing outcome measures

| Study, year (reference)^a^ | | Study center | Study design | Setting | Patients prealerting (n) | Patients postalerting (n) |
| --- | --- | --- | --- | --- | --- | --- |
| **Hospital length of stay** | | | | | | |
|  | Arabi et al, 2017 A [20] | Single center 900-bed tertiary care academic hospital | Pre-post implementation study (full text) | ED | 436 | 195 |
|  | Arabi et al, 2017 B [20] | Single center 900-bed tertiary care academic hospital | Pre-post implementation study (full text) | ED | 436 | 699 |
|  | Austrian et al, 2017 [32] | Single center | Pre-post implementation study (full text) | ED | 838 | 1306 |
|  | Guirgis et al, 2017 [21] | Single center 696-bed level 1 trauma, 142 Intensive Care Beds | Retrospective pre-post implementation study (full text) | Hospital-wide sepsis alert program | 1637 | 1568 |
|  | Hayden et al, 2016 A [23] | Single center, academic, annual volume 48,000 | Retrospective pre-post implementation study (full text) | ED | 108 | 130 |
|  | Hayden et al, 2016 B [23] | Single center, academic, annual volume 48,000 | Retrospective pre-post implementation study (full text) | ED | 13 | 32 |
|  | McRee et al, 2017 [34] | Single center | Retrospective pre-post implementation study (full text) | medicine patients (intervention vs control units) | 75 | 96 |
|  | Sawyer et al, 2011 [28] | Single center, 1250-bed, academic | Prospective observational pre-post implementation study (full text) | 6 medicine wards (2 intervention wards, 4 controls) | 181 | 89 |
|  | Umscheid et al, 2015 [26] | Multicenter, 3 hospitals, total 1500 beds, annual volume 70,000 | Pre-post implementation study (full text) | Adult acute inpatient units | 594 | 545 |
|  | Westra et al, 2017 [25] | Single center, 941 beds, annual volume 42,000 | Pre-post implementation study (full text) | 2 respiratory care units and 1 medical surgical unit | 566 | 212 |
|  | Total | | | | 3948 | 4872 |
| **Intensive care unit length of stay** | | | | | | |
|  | Arabi et al, 2017 A [20] | Single center 900-bed tertiary care academic hospital | Pre-post implementation study (full text) | ED | 436 | 195 |
|  | Arabi et al, 2017 B [20] | Single center 900-bed tertiary care academic hospital | Pre-post implementation study [full text] | ED | 436 | 699 |
|  | Austrian et al, 2017 [32] | Single center | Pre-post implementation study (full text) | ED | 838 | 1306 |
|  | Guirgis et al, 2017 [21] | Single center 696-bed level 1 trauma, 142 intensive care beds | Retrospective pre-post implementation study (full text) | Hospital-wide sepsis alert program | 1637 | 1568 |
|  | Hayden et al, 2016 A [23] | Single center, academic, annual volume 48,000 | Retrospective pre-post implementation study (full text) | ED | 108 | 130 |
|  | Hayden et al, 2016 B [23] | Single center, academic, annual volume 48,000 | Retrospective pre-post implementation study (full text) | ED | 13 | 32 |
|  | Umscheid et al, 2015 [26] | Multicenter, 3 hospitals, total 1500 beds, annual volume 70,000 | Pre-post implementation study (full text) | Adult acute inpatient units | 595 | 545 |
|  | Total | | | | 3627 | 4475 |
| **Time to antibiotics** | | | | | | |
|  | Arabi et al, 2017 A [20] | Single center 900-bed tertiary care academic hospital | Pre-post implementation study (full text) | ED | 436 | 195 |
|  | Arabi et al, 2017 B [20] | Single center 900-bed tertiary care academic hospital | Pre-post implementation study (full text) | ED | 436 | 699 |
|  | Crum et al, 2013 [29] | Single center, academic, level 1 trauma center | Pre-post implementation study (abstract only) | ED | 98 | 103 |
|  | Hayden et al, 2016 A [23] | Single center, academic, annual volume 48,000 | Retrospective pre-post implementation study (full text) | ED | 108 | 130 |
|  | Hayden et al, 2016 B [23] | Single center, academic, annual volume 48,000 | Retrospective pre-post implementation study (full text) | ED | 13 | 32 |
|  | Narayanan et al, 2016 [30] | Single center, academic | Pre-post implementation observational study (full text) | ED | 111 | 103 |
|  | Pulia et al, 2016 [33] | Single center | Pre-post implementation study (abstract only) | ED | 225 | 211 |
|  | Total | | | | 991 | 1473 |
| **Mortality** | | | | | | |
|  | Arabi et al, 2017 A [20] | Single center 900-bed tertiary care academic hospital | Pre-post implementation study (full text) | ED | 436 | 195 |
|  | Arabi et al, 2017 B [20] | Single center 900-bed tertiary care academic hospital | Pre-post implementation study (full text) | ED | *436* | 699 |
|  | Austrian et al, 2017 [32] | Single center | Pre-post implementation study (full text) | ED | 838 | 1306 |
|  | Benson et al, 2014 [22] | Single center 350-bed teaching hospital | Retrospective pre-post implementation study (full text) | All medical/surgical wards | 123 | 116 |
|  | Berger et al, 2010 [31] | Single center tertiary care level 1 trauma center, annual volume 70,000 | Pre-post implementation study (full text) | ED | 908 | 890 |
|  | Ferreras et al, 2015 [27] | Single center | Retrospective pre-post implementation study (full text) | ED | 117 | 116 |
|  | Guirgis et al, 2017 [21] | Single center 696-bed level 1 trauma, 142 intensive care beds | Retrospective pre-post implementation study (full text) | Hospital-wide sepsis alert program | 1637 | 1568 |
|  | Hayden et al, 2016 A [23] | Single center, academic, annual volume 48,000 | Retrospective pre-post implementation study (full text) | ED | 108 | 130 |
|  | Manaktala et al, 2017 [24] | Single center, 941 beds, annual volume 42,000 | Pre-post implementation study (full text) | 2 respiratory units and 1 general medicine unit | 566 | 212 |
|  | Mathews et al, 2014 [35] | Single center | Pre-post implementation study (abstract only) | medicine patients (intervention vs control units) | 879 | 1212 |
|  | McRee et al, 2017 [34] | Single center | Retrospective pre-post implementation study (full text) | medicine patients (intervention vs control units) | 75 | 96 |
|  | Sawyer et al, 2011 [28] | Single center, 1250 beds, academic | Prospective observational pre-post implementation study (full text) | 6 medicine wards (2 intervention wards and 4 controls) | 181 | 89 |
|  | Total | | | | 5868 | 6629 |

^a^The studies by Arabi et al [20] and Hayden et al [23] had 2 separate cohorts of patients.

ED = Emergency Department
