# Supplementary material for: Digital Alerting and Outcomes in Patients With Sepsis: Systematic Review and Meta-Analysis
Source: J Med Internet Res. 2019 Dec 20;21(12):e15166. doi: 10.2196/15166 (PMC6942184; doi:10.2196/15166)
Supplement: Multimedia Appendix 5 [file jmir_v21i12e15166_app5.docx]

Multimedia appendix table 3. Diagnostic accuracy.

| Study | Sensitivity (%), 95% CI | Specificity (%), 95% CI | Positive predictive value (%), 95% CI | Negative predictive value (%), 95% CI | Likelihood ratio (%), 95% CI | Positive likelihood ratio (%), 95% CI | Negative likelihood ratio (%), 95% CI |
| --- | --- | --- | --- | --- | --- | --- | --- |
| Ferreras et al, 2015 [27] | 87 (81.93-91.66) | 94.56 (93.64-95.32) | 51.64 (46.15-57.11) | 99.11 (98.69-99.4) | Not given | Not given | Not given |
| Manaktala et al, 2017 [24] | 95.16 (89.77-98.20) | 81.92 (78.73-84.8) | 50.21 (43.64-56.78) | 98.88 (97.58-99.59) | Not given | 5.26 (4.45-6.23) | 0.06 (0.03-0.13) |
| Sawyer et al, 2011 [28] | Not given | Not given | 19.5 (quoted from another reference and not found in this paper) | 95.8 (quoted from another reference and not found in this paper) | Not given | Not given | Not given |
| Umscheid et al, 2015 [26] | 16 (alert group) 17 (validation group) | 97 (alert group) 97 (validation group) | 26 (alert group) 28 (validation group) | 94 (alert group) 95 (validation group) | 5.3 (alert group) 5.7 (validation group) | Not given | Not given |
| Westra et al, 2017 [25] | 95.2 | 82.0 | 50.6 | Not given | Not given | Not given | Not given |
