# Supplementary material for: Digital Alerting and Outcomes in Patients With Sepsis: Systematic Review and Meta-Analysis
Source: J Med Internet Res. 2019 Dec 20;21(12):e15166. doi: 10.2196/15166 (PMC6942184; doi:10.2196/15166)
Supplement: Multimedia Appendix 6 [file jmir_v21i12e15166_app6.docx]

Multimedia appendix table 4. Risk of bias of nonrandomized prospective comparative cohort comparing pre and postalerting based on the Newcastle-Ottawa Scale.

| Study | Selection: representative of exposed cohort | Selection: representative of control cohort | Ascertainment of exposure | Absence of outcome at start of study | Comparability: study controls for study design | Comparability: study controls for analysis | Assessment of outcome | Duration of follow-up | Adequacy of follow-up | Total score |
| --- | --- | --- | --- | --- | --- | --- | --- | --- | --- | --- |
| Arabi et al, 2017 [20] | 1 | 1 | 1 | 1 | 1 | 1 | 1 | — | — | 7 (higher) |
| Austrian et al, 2017 [32] | 1 | 1 | 1 | 1 | 1 | 1 | 1 | — | — | 7 (higher) |
| Benson et al, 2014 [22] | 1 | 1 | 1 | 1 | — | — | 1 | — | — | 5 (lower) |
| Berger et al, 2010 [31] | 1 | 1 | 1 | 1 | — | 1 | — | — | — | 5 (lower) |
| Crum et al, 2013 [29] | — | 1 | 1 | 1 | — | — | 1 | — | — | 4 (lower) |
| Ferreras et al, 2015 [27] | 1 | 1 | 1 | — | — | — | 1 | — | — | 4 (lower) |
| Guirgis et al, 2017 [21] | 1 | 1 | 1 | 1 | 1 | 1 | 1 | — | — | 7 (higher) |
| Hayden et al, 2016 [23] | 1 | 1 | 1 | 1 | 1 | 1 | 1 | — | — | 7 (higher) |
| Manaktala et al, 2017 [24] | 1 | 1 | 1 | 1 | 1 | 1 | 1 | — | — | 7 (higher) |
| Mathews et al, 2014 [35] | 1 | 1 | 1 | 1 | — | — | 1 | — | — | 5 (lower) |
| McRee et al, 2017 [34] | — | 1 | 1 | 1 | 1 | 1 | 1 | — | — | 6 (lower) |
| Narayanan et al, 2016 [30] | — | 1 | 1 | 1 | 1 | 1 | 1 | — | — | 6 (lower) |
| Pulia et al, 2016 [33] | — | 1 | 1 | 1 | — | 1 | 1 | — | — | 5 (lower) |
| Sawyer et al, 2011 [28] | — | 1 | 1 | 1 | — | 1 | 1 | — | — | 5 (lower) |
| Umscheid et al, 2015 [26] | — | 1 | 1 | 1 | — | 1 | 1 | — | — | 5 (lower) |
| Westra et al, 2017 [25] | 1 | 1 | 1 | 1 | 1 | 1 | 1 | — | — | 7 (higher) |

Footnote

- = missing data
